# Supplementary material for: DYN-1 regulates SPD-2 and PLK-1 localization and mitotic spindle pole organization
Source: Mol Biol Cell. 2026 Mar 18;37(4):br10. doi: 10.1091/mbc.E25-07-0337 (PMC13008268; doi:10.1091/mbc.E25-07-0337)
Supplement: Supplementary file 1 [file mbc-37-br10-s001.pdf]

Supplemental Materials

Molecular Biology of the Cell

Dierlam *et al*.

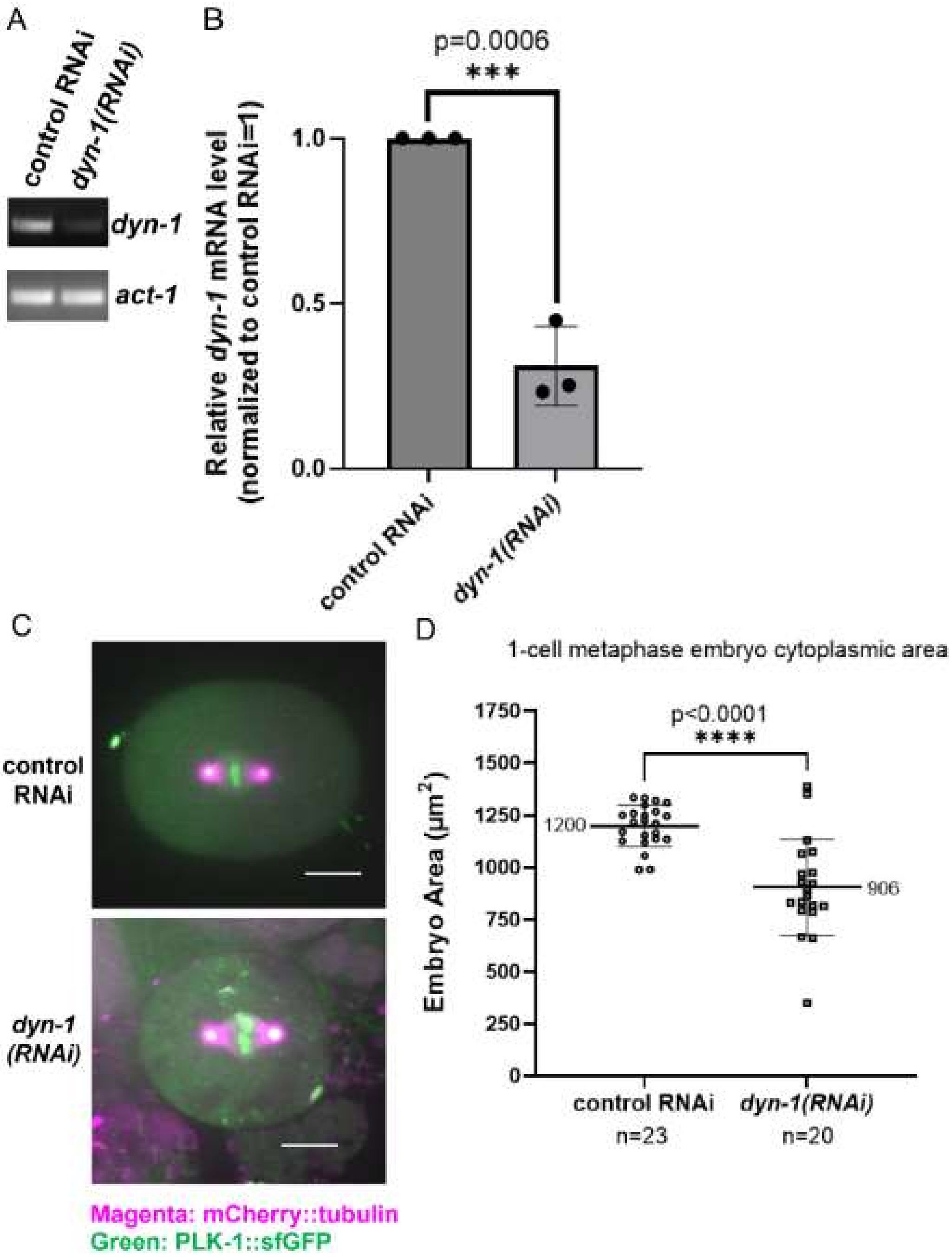

FIGURE S1. *dyn-1(RNAi)* reduces *dyn-1* mRNA levels and decreases embryo size. (A) RT-PCR validation of *dyn-1* depletion. (B) Relative *dyn-1* mRNA quantification. Error bars = s.d. (C) Comparison of average embryo sizes in control RNAi and *dyn-1(RNAi)*. (D) Cytoplasmic area

quantification using PLK-1::sfGFP fluorescence as a marker for the cytoplasmic embryo border. n = embryos. Error bars = s.d.; middle bar = mean. (B, D): unpaired two-tailed t-test; Experimental replicates: 3 for all assays. All fluorescence images include a 10 µm scale bar.

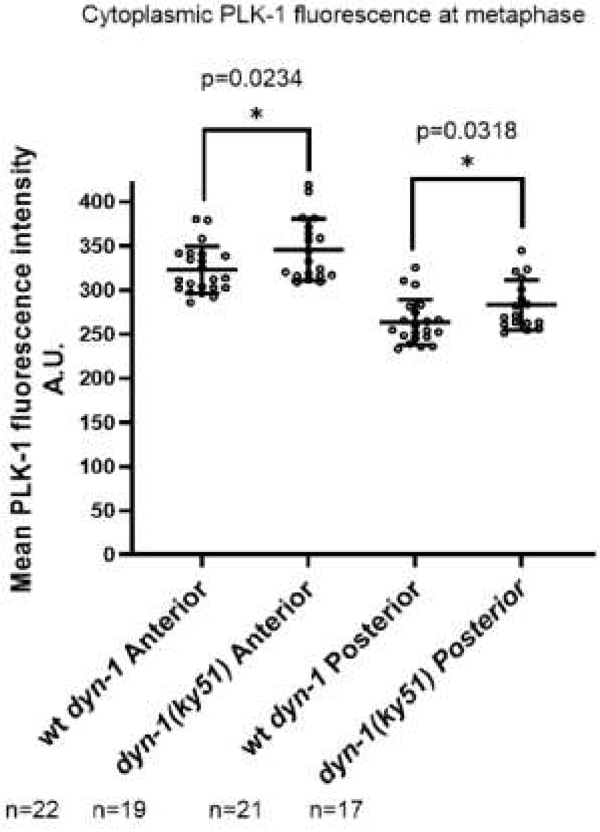

FIGURE S2. Cytoplasmic PLK-1::sfGFP levels are modestly elevated in *dyn-1(ky51)* embryos as compared to wild-type. (A) Quantification of anterior and posterior cytoplasmic PLK-1::sfGFP in wild-type (wt) *dyn-1* and *dyn-1(ky51)* embryos. A.U.= Arbitrary Units; unpaired two-tailed t-test; n = measurements. Error bars = s.d.; middle bar = mean. Experimental replicates: 3.

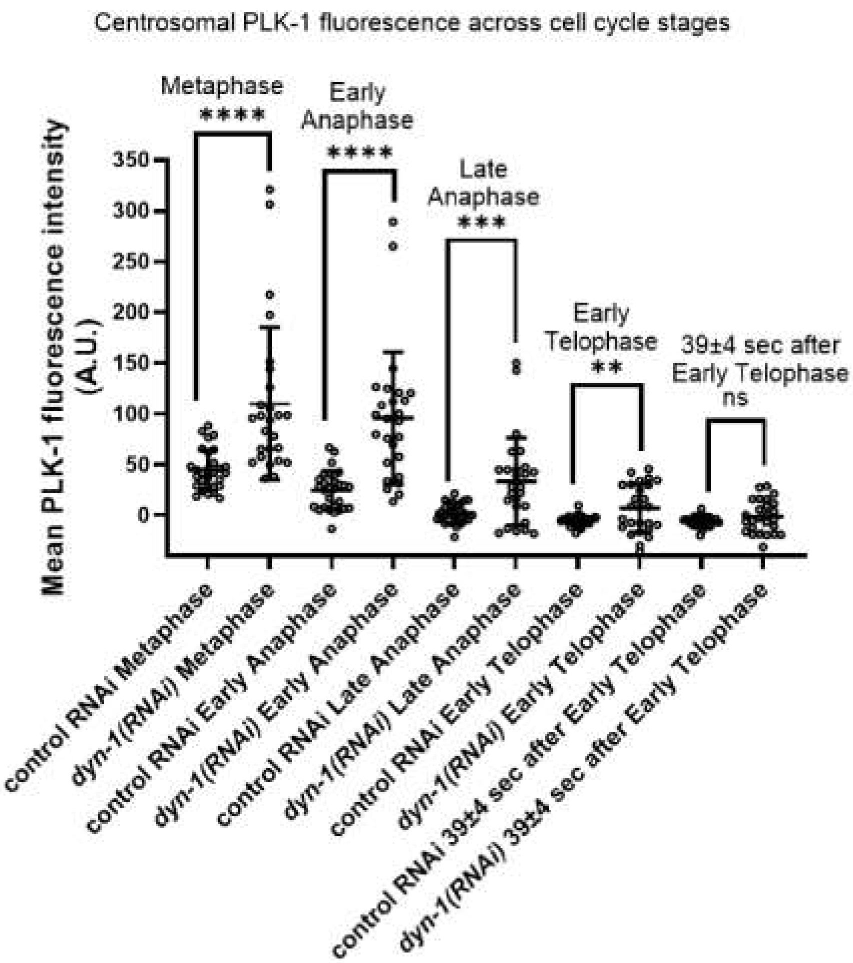

FIGURE S3. Comparison of centrosomal PLK-1 intensity across cell cycle stages in control RNAi and *dyn-1(RNAi)* embryos. (A) Centrosomal PLK-1::sfGFP intensity at metaphase, early and late anaphase, early telophase, and 39±4 seconds post early telophase. A.U.= Arbitrary Units; unpaired two-tailed t-test; n = 29 centrosomes (control RNAi); n = 26 centrosomes (*dyn-1(RNAi))*. Error bars = s.d.; middle bar = mean. Experimental replicates: 3.
